# Annotating targets with pathways: extending approaches to mode of action analysis

**DOI:** 10.1186/1758-2946-5-S1-P15

**Published:** 2013-03-22

**Authors:** Sonia Liggi, Alexios Koutsoukas, Yasaman Kalantar Motamedi, Robert C Glen, Andreas Bender

**Affiliations:** 1 Unilever Centre for Molecular Informatics, Department of Chemistry, University of Cambridge, Cambridge, CB2 1EW, UK

## 

When attempting to treat pathological conditions, it is often necessary to act on multiple targets in order to modify the implicated biological network(s). A systems biology approach would help the drug discovery field by providing a link between chemistry and biology [1]. In particular, annotation of targets with pathways would allow a better understanding of a drug mechanism of action, side effects [2] and promiscuity [3], as well as complex network modulation [4]. A deep understanding of these factors is fundamental to design a drug with the desired pharmacological profile against multiple targets and, to the extent possible, to avoid side effects.

In this context, a cheminformatics target prediction tool [5] was used on several small molecule phenotypic datasets (such as cytotoxicity), and the predicted targets were annotated with the pathways they belong to. Predicted targets and annotated pathways were subjected to an enrichment calculation in order to eliminate the prediction noise and highlight only those likely to be implicated in the phenotype studied. Several enrichment methods were tested and compared, leading to the conclusion that there is not a single method that stands out, but instead the combination of several methods is needed to increase the significance of the readouts.

This protocol allowed us to highlight pathways implicated in the analysed phenotypes which would not be identified by considering only predicted targets, hence giving further insight into the mechanism of expression of the observed phenotype.
